# Maternal Donor and Genetic Variation of *Lagerstroemia indica* Cultivars

**DOI:** 10.3390/ijms24043606

**Published:** 2023-02-10

**Authors:** Chang Guo, Kangjia Liu, Enze Li, Yifeng Chen, Jiayao He, Wenying Li, Wenpan Dong, Zhili Suo

**Affiliations:** 1 Laboratory of Systematic Evolution and Biogeography of Woody Plants, School of Ecology and Nature Conservation, Beijing Forestry University, Beijing 100083, China; 2 School of Forestry, Beijing Forestry University, Beijing 100083, China; 3 Institute of Ecological Conservation and Restoration, Chinese Academy of Forestry, Beijing 100091, China; 4 State Key Laboratory of Systematic and Evolutionary Botany, Institute of Botany, Chinese Academy of Sciences, Beijing 100093, China

**Keywords:** *Lagerstroemia indica*, crape myrtles, genetic variation, plastome, nuclear ribosomal DNA

## Abstract

*Lagerstroemia indica* L. is a well-known ornamental plant with large pyramidal racemes, long flower duration, and diverse colors and cultivars. It has been cultivated for nearly 1600 years and is essential for investigating the germplasm and assessing genetic variation to support international cultivar identification and breeding programs. In this study, 20 common *Lagerstroemia indica* cultivars from different varietal groups and flower morphologies, as well as multiple wild relative species, were analyzed to investigate the maternal donor of *Lagerstroemia indica* cultivars and to discover the genetic variation and relationships among cultivars based on plastome and nuclear ribosomal DNA (nrDNA) sequences. A total of 47 single nucleotide polymorphisms (SNPs) and 24 insertion/deletions (indels) were identified in the 20 *L. indica* cultivars’ plastome and 25 SNPs were identified in the nrDNA. Phylogenetic analysis based on the plastome sequences showed that all the cultivars formed a clade with the species of *L. indica*, indicating that *L. indica* was the maternal donor of the cultivars. Population structure and PCA analyses supported two clades of cultivars, which exhibited significant genetic differences according to the plastome dataset. The results of the nrDNA supported that all 20 cultivars were divided into three clades and most of the cultivars had at least two genetic backgrounds and higher gene flow. Our results suggest that the plastome and nrDNA sequences can be used as molecular markers for assessing the genetic variation and relationships of *L. indica* cultivars.

## 1. Introduction

Crape myrtles (*Lagerstroemia indica* L.), belonging to the genus *Lagerstroemia* of the family Lythraceae, are important summer-blooming ornamental trees or shrubs. They are excellent woody plants for environmental protection, since they can absorb smoke and dust in the air and are resistant to toxic gases such as sulfur dioxide, hydrogen fluoride, and chlorine released from industrial pollution [1]. Crape myrtles are valuable, with high economic value in city gardening and hill planting. There are more than 200 cultivars of *L. indica* in the world, and crape myrtles have been cultivated for nearly 1600 years [1,2]. The breeding of new cultivars with various outstanding features, such as a longer flowering time; different floral colors; stronger aroma; seeds with higher oil production; or resistance to drought, coldness, insects, or disease, is an important issue.

Morphological characteristics, such as flower color, length and width of inflorescence, flower diameter, floral fragrance, number of flowerlets per inflorescence, petal claw color, leaf color, and thousand-seed weight, are major elements for understanding phenotypic differentiation in crape myrtle cultivars. The complex genetics and the high phenotypic variation of *L. indica* cultivars are thought to be the result of multiple cycles of artificial selection and intraspecific hybridization [3,4]. Intraspecific hybridization is mainly used for cultivar improvement, with selection of the parent cultivar a critical step in hybrid breeding. Phenotypic traits are frequently used as the main guide for choosing the germplasm to develop new cultivars with novel appearances and improved stress tolerance. In fact, selecting the germplasm with relatively large genetic differences can help improve breeding efficiency, as hybrid breeding according to phenotype may not produce offspring with the expected characteristics. Understanding the genetic background of *L. indica* cultivars is essential for choosing germplasm resources and ultimately facilitating genetic improvement and breeding programs.

The origin of cultivated crape myrtles has attracted great attention over the past decades. Previous studies show that the donor of cultivated crape myrtles involved five *Lagerstroemia* species based on the morphological and genetic data, including *L. indica, L. fauriei*, *L. speciosa*, *L. subcostata,* and *L. limii* [3,5]. However, the maternal donor of the cultivated crape myrtles is not clear. Morphological characteristics [6], RAPD [7], AFLP [8], and SSRs [2,9,10,11,12] markers were used to assess genetic diversity among the *L. indica* cultivars. However, few studies have evaluated the genetic variation and diversity of *L. indica* cultivars using DNA sequence markers. Improved DNA sequence markers need to be developed to facilitate research access to the genetic diversity of crape myrtles.

Plastome and nuclear ribosomal DNA (nrDNA) sequences are popular molecular markers used for plant phylogeny [13,14,15,16,17] and species identification [18,19] because these sequences are conserved across plant species and show variability among interspecies levels. Past studies show that the plastome and nrDNA sequences contain numerous SSRs, indels, and SNPs at the intraspecies levels, which have been used to research the genetic divergence of endangered species [20,21], biogeographical structure [22,23,24], gene flow among subpopulations [25,26], and origins and domestication of cultivars [27,28,29]. For the *Lagerstroemia* species, more than 17 species have sequenced the whole chloroplast genome, using these data to infer the phylogeny and divergence time of *Lagerstroemia* [30]. Dong et al. [30] also identified several polymorphism sites in the plastome and nrDNA sequences at the intraspecies level in *L. indica*, indicating that these molecular data will resolve genetic variation among the *L. indica* cultivars at the genome level.

In this study, we performed comprehensive sampling in cultivars of *L. indica*. A total of 20 accessions were collected to represent different varietal groups and flower morphologies [6,31]. All of the plastome and nrDNA sequences were assembled to discover the sequence variation among the cultivars. Phylogenetic analyses combining data of the cultivars and of wild species elucidated the relationships between and maternal origin of crape myrtle cultivars. Genetic diversity and population differentiation analyses evaluated the genetic structure and genetic divergence in the cultivars. This study sheds light on the diversity of crape myrtle cultivars and provides variable genetic resources for the breeding of new cultivars.

## 2. Results

### 2.1. Plastome and nrDNA Sequences of Lagerstroemia indica Cultivars

**Figure 1:**
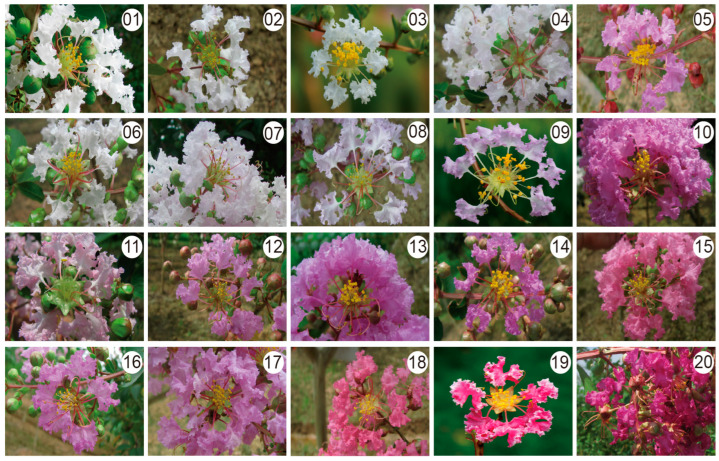
The floral traits of 20 *Lagerstroemia indica* cultivars. 01. ‘Baimixiang’; 02. ‘Baiyunyingxia’; 03. ‘Bingqingyudie’; 04. ‘Zizhuainwei’; 05. ‘Dahuazhaolu’; 06. ‘Xiaohuayinwei’; 07. ‘Dahuaziyun’; 08. ‘Zhoubanjinwei’; 09. ‘Dahuacuipanjinwei’; 10. ‘Duohuazi’; 11. ‘Qiaojiaren’; 12. ‘Jinwei’; 13. ‘Lanzi’; 14. ‘Duohuajinxiu’; 15. ‘Fenjing’; 16. ‘Ziyu’; 17. ‘Zixia’; 18. ‘Caixiamantian’; 19. ‘Yinbianhong’; 20. ‘Hongzhuashenzi’.

In this study, the plastomes of 20 cultivars of *L. indica* were assembled (Figure 1 and Table 1). All the plastomes had the typical quadripartite structure of most angiosperm plants. The length of these plastomes varied between 152,174 bp and 152,232 bp, with the LSC (length: 84,006 bp–84,062 bp) and SSC (length: 16,908 bp–16,910 bp) separated by two IRs (length: 25,630) (Table S2). The overall GC content was 37.6%. The *L. indica* plastome harbored 112 different genes, including 78 protein coding genes, 30 tRNA genes, and 4 rRNA genes. The annotated plastomes were deposited in GenBank (Table 1). The positions of the IR and SC boundaries were conserved among the cultivars. The LSC and IRb boundary was located in the *rps19* gene, and the IRb and SSC in the *ndhF* gene. The boundary between LSC and IRa was located between the *rps19* and *trnH-GUG*. The *trnH-GUG* gene was located at the beginning of the LSC region. The nrDNA sequences were each assembled into a single contig using the GetOrganelle toolkit. The nrDNA sequences were aligned with 6419 bp.

### 2.2. Plastome Variation in the Lagerstroemia indica Cultivars

**Figure 2:**
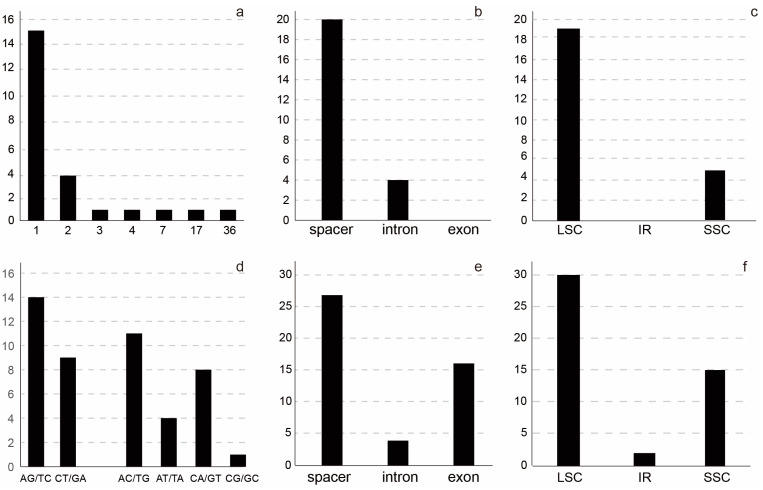
Plastome variation in the *Lagerstroemia indica* cultivars. (**a**) length of indels, *X*-axis indicated the length of the indels; (**b**) number of indels in the spacer, intron, and exon regions; (**c**) number of indels in the LSC, IR, and SSC regions; (**d**) patterns of SNPs, X-axis indicated the patterns of the SNPs; (**e**) number of SNPs in the spacer, intron, and exon regions; (**f**) number of SNPs in the LSC, IR, and SSC regions.

The *L. indica* cultivars’ plastomes were aligned with 152,250 bp in length. Indels and SNPs were identified in the plastomes, and most of the intraspecific *L. indica* variable sites and indels were located in the LSC and SSC regions (Figure 2). A total of 24 indels were discovered in the 20 *L. indica* cultivars’ plastomes, including 14 SSR-related indels, 4 repeat-related indels, and 6 normal indels. All the indels occurred in the noncoding regions, including 4 in introns (*ndhA*, *clpP*, *atpF*, and *petB*) and 20 in the spacer regions (Figure 2c). The indels’ size ranged from 1 to 36 bp (Figure 2a), with 1 bp indels occurring in the highest frequency (62.5%). The two largest indels were located in the *rpl33-rps18* and *accD-psaI* regions, both of which were repeat-related. Both of these indels were found in an insert in the ‘Qiaojiaren’, ‘JinWei’, ‘Zhoubanjinwei’, ‘Baimixiang’, and ‘Lanzi’ cultivars.

There were 47 SNPs in the 20 *L. indica* plastomes, and the average number of intraspecific variable sites was 0.31 per kb. Among the 47 SNPs, there were 45 parsimony-informative sites, including 23 transition (Ts) and 24 transversion (Tv) sites. The most frequent SNP mutation types were A to G and T to C, with G to C or C to G mutations occurring far less frequently, and only once in the *psbE-petL* region. A total of 30, 15, and 2 SNPs occurred in the LSC, SSC, and IR regions, respectively. SNPs were harbored in 38 sequence regions, including 23 spacer regions, 12 coding regions, and 4 intron regions. The *trnK-UUU-rps16* spacer region and the *ycf1* gene had three SNPs; *trnD-GUC-trnY-GUA*, *rpl32-trnL-UAG*, *rpoC2*, *ndhD*, and *ndhF* had two SNPs, and the rest of the regions had one SNP.

### 2.3. Nuclear Ribosomal DNA Variability

The nrDNA sequences were highly homogeneous among the 20 *L. indica* cultivars, with an aligned length of 6,419 bp. The GenBank accession numbers of the nrDNA of the cultivars are shown in Table 1. Comparison of the sequences revealed 25 SNPs: 11 in the 18S rRNA region, 11 in the ITS region, and 3 in the 26S region.

### 2.4. Maternal Origin of the Cultivars

**Figure 3:**
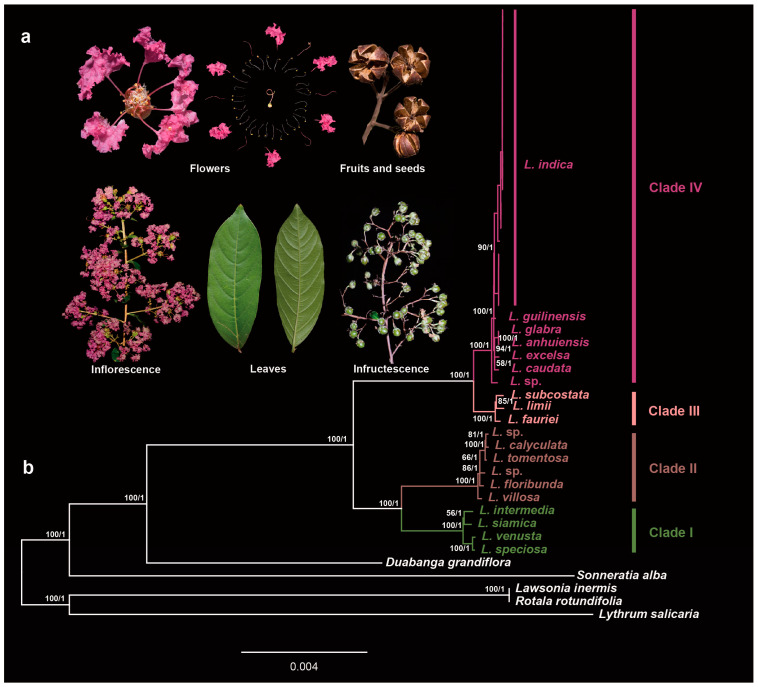
Phylogenetic relationships between *Lagerstroemia indica* and other related wild species. (**a**) morphological characteristics of *Lagerstroemia indica*; (**b**) ML bootstrap support values/Bayesian posterior probabilities are shown at each node.

Combining the wild species and cultivars, ML and BI analyses based on the whole plastome dataset produced similar trees (Figure 3). The dataset strongly supported the monophyly and revealed four clades in the genus *Lagerstroemia*. This result is consistent with recent phylogenetic results [30,32]. All 20 cultivars of *L. indica* formed a strongly supported group with the wild species of *L. indica*, with higher supported values (BS/PP = 90/1) in clade IV indicating that the maternal parentage of all 20 cultivars was the *L. indica*.

### 2.5. Genetic Variation Based on the Plastome Sequences

**Figure 4:**
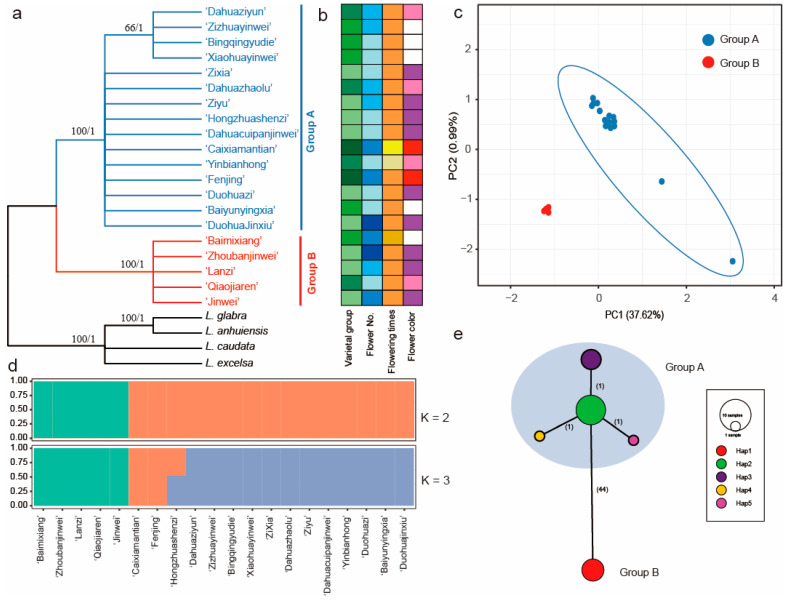
Intraspecific diversity and genetic structure of 20 *Lagerstroemia indica* cultivars based on plastome dataset. (**a**) phylogenetic tree. ML bootstrap support values/Bayesian posterior probabilities are shown at each node; (**b**) phenotypic characterization; (**c**) principal component analysis; (**d**) population structure analysis with K = 2, 3; (**e**) TCS network of five haplotypes from the plastome sequences. The number of mutational steps is shown on the lines, and the size of the pie chart represents the number of the accessions. The haplotype for each sample is listed in Table S3.

ML and BI tree analyses performed from the whole plastome sequences indicated that all 20 cultivars of *L. indica* formed two clades (Figure 4a). The five cultivars of ‘Qiaojiaren’, ‘JinWei’, ‘Zhoubanjinwei’, ‘Baimixiang’, and ‘Lanzi’ formed a clade. The PCA scatterplot is presented in Figure 4c. The first two PCA axes account for about 38.61%, revealing a clear clustering in the two groups. Population structure results from ADMIXTURE suggest that there are two clades (Figure 4d and Figure S1).

All the polymorphisms allowed for the identification of five haplotypes (Figure 4e, Table S3). The five haplotypes also formed two clades, exhibiting a significant genetic difference with the number of mutational steps (44 steps). Further evidence of the phylogenetic structure of plastome variation in the *L. indica* cultivars was provided by the distribution of SNP variation among the phylogenetic clades. Haplotype 1 contained five cultivars of ‘Qiaojiaren’, ‘JinWei’, ‘Zhoubanjinwei’, ‘Baimixiang’, and ‘Lanzi’. Haplotype 2, containing nine cultivars, formed a clade with haplotypes 3, 4, and 5, showing a star-like topology consisting of a central haplotype (hap2) from which the other three haplotypes radiate, separated by one step (Figure 4e).

### 2.6. Genetic Variation Based on the nrDNA Sequences

**Figure 5:**
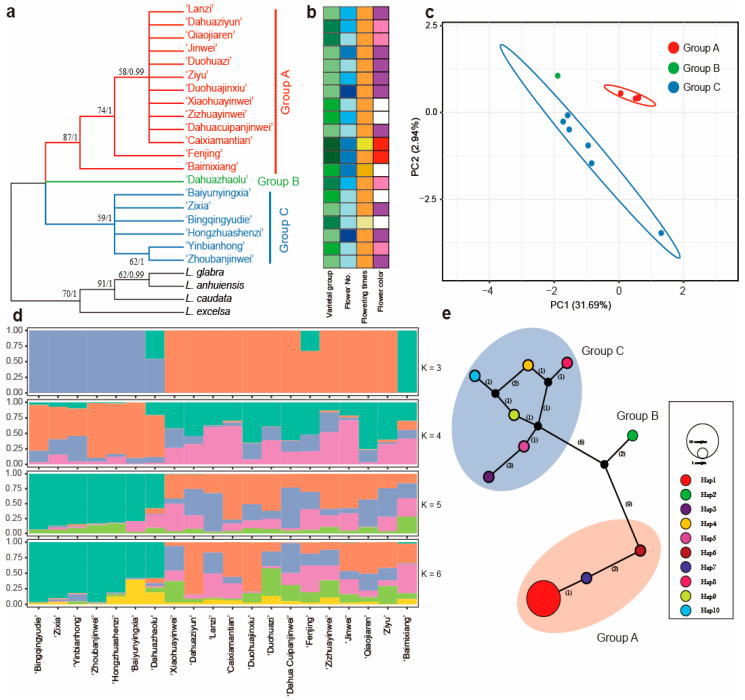
Intraspecific diversity and genetic structure of 20 *Lagerstroemia indica* cultivars based on nrDNA dataset. (**a**) phylogenetic tree. ML bootstrap support values/Bayesian posterior probabilities are shown at each node; (**b**) phenotypic characterization; (**c**) principal component analysis; (**d**) population structure analysis with K = 3, 4, 5, and 6; (**e**) TCS network of 10 haplotypes from the nrDNA sequences. The number of mutational steps is shown on the lines, and the size of the pie chart represents the number of the accessions. The black circles are extinct haplotypes, and the haplotype for each sample is listed in Table S4.

Phylogenetic reconstruction using the nrDNA sequences revealed three clades dividing the twenty cultivars of *L. indica* (Figure 5a). The PCA scatterplot is presented in Figure 5c. The first two PCA axes account for about 34.63%, revealing a clear clustering in the three groups. Population structure was analyzed using K values ranging from 1 to 10, and the cross validation (CV) error was also the lowest with K = 5 (Figure 5d and Figure S1). The results show that most of the cultivars had at least two genetic backgrounds and had higher gene flow.

Network analysis supported three clades, consistent with the phylogeny result (Figure 5e). Ten haplotypes were identified (Table S4), with only haplotype 1 (hap 1) represented across eleven cultivars. With haplotype 2, haplotypes 1, 6, and 7 formed a clade (Group A in Figure 5a) with nine mutational steps. Haplotype 2 was the ‘Dahuazhaolu’ from the Bicolor group. The population structure shows ‘Dahuazhaolu’ with multiple instances of crossbreeding (Figure 5d). Group C included six haplotypes, containing six cultivars of ‘Yinbianhong’, ‘Bingqingyudie’, ‘Zixia’, ‘Baiyunyingxia’, Hongzhuashenzi’, and ‘Zhoubanjinwei’.

### 2.7. Phenotypic Characterization

The 20 cultivars of *L. indica* showed very high morphological variation (Figure 1). The different varietal groups exhibited variation in flower numbers and flowing time, and neither the plastome nor nrDNA data supported a finding that the varietal groups were monophyletic. (Figure 4b and Figure 5b). There were four orders of flower numbers in the inflorescence of the twenty cultivars, showing greater differences. The two cultivars of ‘Duohuajinxiu’ and ‘Zhoubanjinwei’ exhibited a large number of flowers (more than 200) per inflorescence, while the ‘Dahuazhaolu’ cultivar from the ‘Bicolor’ group only had half that amount. Most of the cultivars (17) bloom in July and August. The flowering time of cultivar ‘Yinbianhong’ is relatively early, before July, while ‘Caixiamantian’ flowers relatively late, in September. The ‘Baimixiang’ cultivar from the ‘Alba’ group has a strong fragrance and a long flowering time (from July to September). Finally, flower color was documented in 20 cultivars (Figure 1). The most common colors are white, purple, red, and bicolor (mostly purple and pink). Flower color is the main varietal group-based phenotypic character for *L. indica* cultivars. However, flower color-based grouping was not supported by molecular data.

## 3. Discussion

### 3.1. Maternal Donor of Crape Myrtle Cultivars

Crape myrtles have been cultivated as ornamental trees for more than 1600 years, owing to their long flowering season, high resistance to pollution, and ease of training. Although crape myrtles have more than 300 cultivars [2], the maternal donor has not historically been clear. In this study, we used whole plastome sequences to explore the maternal donor of crape myrtle cultivars and assess their genetic variation.

The five species of *L. indica*, *L. fauriei*, *L. speciosa*, *L. subcostata,* and *L. limii* have been introduced in crape myrtle breeding programs, releasing at least 200 varieties with a wide range in plant size, habitat, flower color, and size [3,5]. *L. fauriei*, from central and southern Japan, has been an important donor of crape myrtle, owing to its strong resistance to mildew, disease, and cold temperatures. *L. speciosa*, native to Australia, Southern New Guinea, India, and the Philippines, is a woody plant growing 25 m high [33] which is widely cultivated as an ornamental tree in tropical and subtropical areas. *L. subcostata* and *L. limii*, native to Southern China, are mostly shrubs or small trees; they bloom earlier [33] and have been used to breed early-flowering cultivars [34].

Cross-breeding is one of the primary strategies for the breeding of crape myrtles, with five main wild species involved in the breeding of its cultivars. Our phylogeny results revealed that all 20 cultivars formed a strongly supported clade within the wild species of *L. indica* (Figure 3), indicating that *L. indica* was the maternal donor of these cultivars. *Lagerstroemia guilinensis*, narrowly distributed in Guangxi Province, is sister to *L. indica* in Clade IV. Phylogeny results supported that the three species of *L. fauriei*, *L. subcostata*, and *L. limii* form a separate clade (Clade III).

### 3.2. Phenotypic Diversity and Genetic Variation of Lagerstroemia indica Cultivars

The *L. indica* cultivars are rich in phenotypic diversity (Figure 1), including quantitative and qualitative trait variations such as flower color, claw color, flower diameter, flower number, length and width of inflorescence, and 1000-seed weight [6,8]. The high degree of phenotypic diversity varies among the different varietal groups; the ‘Rubra’ group had the highest phenotypic diversity, followed by the ‘Amabilis’ group and the ‘Alba’ group [6]. Based on the cluster of phenotypic characteristics, the *L. indica* cultivars were further divided into five groups, and all the varietal groups did not form a clade except the ‘Bicolor’ group [6]. Meanwhile, genetic evidence did not support the finding of a monophyletic group within the varietal groups from either the plastome or nrDNA sequences (Figure 4a and Figure 5a). Phylogenetic relationships showed there was conflict between plastome and nrDNA datasets (Figure 4a and Figure 5a). Topological cytonuclear discordance is commonly observed in plant phylogenetics [14,35], and incomplete lineage sorting and gene flow can cause cytonuclear discordance within the species or among closely related species [36,37]. For *L. indica* cultivars, the intraspecific hybridization during the process of cross-breeding may lead to the discordance between the plastome and nrDNA. Plastome and nrDNA sequencing revealed high genetic diversity among crape myrtle cultivars. The plastome sequences unveiled unexplored genetic variation, and 47 variable sites and 24 indels were identified, giving rise to 5 haplotypes and dividing the 20 cultivars into 2 groups (Figure 4). The nrDNA sequence included 25 variable sites and divided the 20 cultivars into 3 clades (Figure 5). The plastome and nrDNA sequences exhibited high variability in crape myrtle cultivars, compared to *Chrysanthemum* [27], sweet potato (*Ipomoea batatas*) [38], and *Panax ginseng* [39] cultivars. Compared to the SSR and AFLP markers, the plastome sequence markers show lower genetic variability; however, as maternal markers, it is essential to identify the maternal parentage.

Further evidence showed that most of the crape myrtle cultivars are of hybrid origin, even interspecific hybrids (for example, some cultivars from America) [5,12]. The structure of the nrDNA also supported the finding that most of the selected cultivars were at least two crosses (Figure 5d). Several studies showed the cultivars mostly tended to group by geographic regions [29]. With wide sampling, the crape myrtle cultivars also showed the same pattern [12]. Interestingly, according to the morphological database, several cultivars exhibited similar traits when forming a clade, such as the same flower color (e.g., ‘Yinbianhong’ and ‘Hongzhuashenzi’). This indicates that they have a similar genetic background (Figure 4 and Figure 5).

### 3.3. Utility of Plastome and nrDNA for Accessing Genetic Diversity of Cultivars

With the advantage of next-generation sequencing technologies and bioinformatics tools, plastomes and nrDNA can be assembled from genome skimming data, avoiding the high experimental technology requirements of chloroplast isolation and purification [40,41,42]. Plastomes are maternally inherited and structurally conserved in most angiosperm plants, and nrDNA sequences are variable, leading these markers to be widely used for tracking the evolution and species identification of plants at both high and low taxonomic levels. Therefore, the plastome markers *rbcL*, *matK*, *ycf1*, and ITS are typically selected as the DNA barcodes for land plants [43,44]. Some of the regions of the plastomes, including the *ndhF*, *trnH-psbA*, and *trnL-F*, have been identified as mutation hotspot regions [45]. For the wild species of *Lagerstroemia*, four variable loci, *trnD-trnY-trnE*, *rrn16-trnI, ndhF-rpl32-trnL*, and *ycf1*, were discovered in the *Lagerstroemia* plastomes [30].

Plastome sequences are widely used to infer phylogenetic relationships at different taxonomic levels. The phylogeny of the species in *Lagerstroemia* was well resolved based on the whole plastome sequences from this paper and from previous studies [30,32,46], revealing the maternal donor of crape myrtle cultivars. Only a few studies have assessed the intraspecific variation of the whole plastomes and nrDNA sequences [18,20,21,47,48]. The plastid genome markers have less use in analyzing the genetic diversity of cultivars, owing to their limited polymorphic sites. In this study, we sequenced the whole plastome and nrDNA of common and representative crape myrtle cultivars to assess the variations in these sequences. In total, 47 SNPs and 24 indels, and 28 SNPs in plastome and nrDNA sequences, respectively, were identified among these 20 crape myrtle cultivars.

Mutation rate variation among different lineages of plastomes and nrDNA sequences has been examined in various studies [49,50,51]. Most of the cultivars originated from one species, and it is difficult to discover the genetic difference owing to these variations occurring at the intra-specific level or even the intra-group level. However, more variable sites were identified in the crape myrtle cultivars. There are three main factors which have the possibility of introducing more genetic variations. First, at least five wild species (*L. indica*, *L. fauriei*, *L. speciosa*, *L. subcostata*, and *L. limii*) are involved in the formation of crape myrtle cultivars [2,3,52], leading to broad genetic variation. Second, most of the cultivars were formed by hybridization, and cross-breeding is one of the primary strategies for the breeding of crape myrtles. Third, longer cultivation and selection based on traits such as flower color and number of flowers per inflorescence have led to the maintenance of more genetic variations.

Additional studies have shown that the plastome has mutational hotspot regions and that the IR region was better conserved than SC regions. In the crape myrtle cultivars’ plastome, intra-specific variable sites and indels were mostly located in the SC regions and the *trnK-UUU-rps16* spacer region. The *ycf1* gene demonstrated higher variability. The nrDNA internal transcribed spacer (ITS) sequences are highly variable in the kingdom Plantae, with a potentially high resolution of inter- and intra-specific relationships [53]. ITS sequences have been used to authenticate ginseng cultivars [54], assess the genetic variability and relationship of banana cultivars (*Musa* L.) [55], and conduct molecular identification of Malaysian pineapple cultivars [56].

## 4. Materials and Methods

### 4.1. Sampling, DNA Extraction, and Sequencing

Twenty *L. indica* cultivars representing different varietal groups and flower morphologies were collected (Figure 1 and Table 1), including all four varietal groups (four cultivars in the ‘Bicolor’ group, five cultivars in the ‘Alba’ group, nine cultivars in the ‘Amabilis’ group, and two cultivars in the ‘Rubra’ group) [1,6,31]. The selected cultivars represented different flower numbers and flowering times (Table 1). We also downloaded the plastome sequences of all the published wild relatives of *L. indica* to elucidate the maternal origin of crape myrtle cultivars.

Total genomic DNA was extracted using the modified CTAB method [57]. DNA quantity and quality were examined by electrophoresis in 1% agarose. Total DNA was sheared by an ultrasonicator to 350 bp fragments, and a paired-end DNA library for Illumina HiSeq X-ten platform sequencing was constructed. Each sample yielded approximately 5 Gb of data.

### 4.2. Plastome and nrDNA Assembly

Trimmomatic 0.36 [58] was used to conduct a quality control of the raw data within the default parameters. Plastome and nrDNA sequences were assembled using the GetOrganelle toolkit [59] with k-mer lengths of 95. If GetOrganelle failed, we used the following method to assemble it: The SPAdes 3.6.1 program (k-mer = 95) [60] was selected to assemble the contigs using the clean data. Plastome contigs, which were selected using the Blast program [61], were manually assembled using Sequencher 5.4.5 (Gene Codes Corporation, Ann Arbor, MI, USA, http://www.genecodes.com, accessed on 10 July 2022). Gaps and assembly errors were filled and checked using the clean reads that were mapped to the contigs using Geneious Prime (Biomatters Ltd., Auckland, New Zealand) [62]. Plastomes were annotated using the perl scripts Plann.pl [63], with the published genome of *L. indica* (GenBank accession number: KX263727) as the reference sequence. Annotation errors and missing genes were checked and manually added with Geneious Prime. Our annotation of plastomes and nrDNA sequences was submitted to GenBank.

### 4.3. Plastome and nrDNA Variation Analyses

All the plastome and nrDNA sequences of the 20 cultivars were aligned using MAFFT 7 [64]. We identified intra-species polymorphism, including SNPs and indel markers. SNPs were identified and calculated using MEGA 7.0 [65], and DnaSP 6 [66] was used to identify the indels. Their number, location, and direction were calculated using the ‘Dahua Zhaolu’ chloroplast genome as the standard reference to determine the mutation direction.

### 4.4. Phylogenetic Analyses

To infer the maternal origins of the 20 *L. indica* cultivars, we combined the plastome data of cultivars with 4 wild *L. indica* samples, 19 other wild *Lagerstroemia* species, and 5 Lythraceae species used as outgroups (Table S1). The plastome and nrDNA of cultivars were also used to infer phylogenetic relationships, with four *Lagerstroemia* species as the outgroups according to the phylogenetic relationships of *Lagerstroemia*. Phylogenetic analyses were performed using the maximum likelihood (ML) and Bayesian inference (BI) methods. For both analyses, the best-fit substitution mode GTR+GAMMA was chosen by ModelFinder [67] under the Bayesian information criterion. ML analysis was conducted in RAxML-NG [68], and the best tree was selected to calculate the node support values using 500 rapid bootstrap replicates.

BI analysis was performed in Mrbayes v3.2 [69]. Markov chain Monte Carlo (MCMC) simulations were run for 10 million generations, with a sampling of 1000 generations. The stationary phase was examined using Tracer 1.6 [70], and the first 25% of the sampled trees were discarded. The majority-rule consensus tree was generated using the remaining trees and estimated posterior probabilities.

### 4.5. Genetic Variation and Diversity Analyses

Plastome and nrDNA sequences of the 20 cultivars were used for structure and PCA analyses. The population structure used the filtered intraspecific SNPs and an admixture model-based clustering method implemented in Admixture v1.3. The optimal number of clusters was evaluated by running the K-means clustering algorithm (K = 1 to K = 10). The most likely number of clusters was determined based on CV error. Principal component analysis (PCA) was conducted using Plink [71], and the ggplot package [72] in R was used to draw the figure. Both of these data were used to perform network analyses. The haplotype data were exported in DnaSP v6 [66], and the haplotype frequencies were performed in Arlequin v3.5 [73]. PopArt v1.7 was used to build the TCS network [74].

### 4.6. Phenotypic Analyses

Four phenotypic characters were used for phenotypic analyses. We analyzed the varietal group according to the classification system of Zhang [1]. The 20 cultivars included four varietal groups. The flower numbers in the inflorescence were divided into four classes: less than 100, 100–150, 150–200, and more than 200. Flowering time was divided into four periods: early flowering (before July), middle flowering (July and August), late flowering (September), and flowering long. The flower color was divided into four groups: white, purple, red, and bicolor (mostly purple and pink).

## 5. Conclusions

In this study, based on plastome and nrDNA, we discovered the genetic variations of crape myrtle (*L. indica*) cultivars and identified genome-wide variances, which contribute to better understanding the origin and relationships of the cultivars. The phylogenetic tree of the plastome, including wild species and cultivars, reveals the maternal origins of cultivars. The structure results of the nrDNA show that most of the cultivars are of hybrid origins. The haplotype identification and phylogeny provide novel insights into the cultivation history of crape myrtle cultivars.

## Figures and Tables

**Table 1:** Samples information and characteristics of the 20 *Lagerstroemia indica* cultivars.

Samples	Varietal Group	Flower Number	Flowering Times	Flower Color	Origin	GenBank Accession Number of Plastome	GenBank Accession Number of nrDNA
‘Dahuazhaolu’	‘Bicolor’ Group	43–98	Middle period (July and August)	Bicolor	Jishou, Hunan	OP613198	OP723643
‘Ziyu’	‘Amabilis’ Group	62–112	Middle period (July and August)	Purple	Changde, Hunan	OP613199	OP723644
‘Duohuazi’	‘Amabilis’ Group	64–172	Middle period (July and August)	Purple	Liuyang, Hunan	OP613200	OP723645
‘Qiaojiaren’	‘Bicolor’ Group	61–138	Middle period (July and August)	Bicolor	Wuhan, Hubei	OP613201	OP723646
‘Zizhuayinwei’	‘Alba’ Group	69–102	Middle period (July and August)	White	Guiyang, Guizhou	OP613202	OP723647
‘Yinbianhong’	‘Bicolor’ Group	86–113	Early flowering period (before July)	Bicolor	Jishou, Hunan	OP613203	OP723648
‘Bingqingyudie’	‘Alba’ Group	76–158	Middle period (July and August)	White	Guiyang, Guizhou	OP613204	OP723649
‘JinWei’	‘Amabilis’ Group	83–185	Middle period (July and August)	Purple	Changsha, Hunan	OP613205	OP723650
‘Zixia’	‘Amabilis’ Group	66–119	Middle period (July and August)	Purple	Changde, Hunan	OP613206	OP723651
‘Zhoubanjinwei’	‘Amabilis’ Group	153–225	Middle period (July and August)	Purple	Changsha, Hunan	OP613207	OP723652
‘Caixiamantian’	‘Rubra’ Group	98–198	Late flowering (September)	Red	Chengdu, Sichuan	OP613208	OP723653
‘Baiyunyingxia’	‘Alba’ Group	57–125	Middle period (July and August)	White	Changsha, Hunan	OP613209	OP723654
‘Baimixiang’	‘Alba’ Group	100–198	Flowering long	White	Changsha, Hunan	OP613210	OP723655
‘Lanzi’	‘Amabilis’ Group	59–106	Middle period (July and August)	Purple	Changde, Hunan	OP613211	OP723656
‘Fenjing’	‘Rubra’ Group	97–198	Middle period (July and August)	Red	Chengdu, Sichuan	OP613212	OP723657
‘Dahuaziyun’	‘Bicolor’ Group	43–122	Middle period (July and August)	Bicolor	Shaoyang, Hunan	OP613213	OP723658
‘Xiaohuayinwei’	‘Alba’ Group	69–145	Middle period (July and August)	White	Chengdu, Sichuan	OP613214	OP723659
‘Duohuajinxiu’	‘Amabilis’ Group	138–222	Middle period (July and August)	Purple	Shaoyang, Hunan	OP613215	OP723660
‘Hongzhuashenzi’	‘Amabilis’ Group	64–164	Middle period (July and August)	Purple	Shaoyang, Hunan	OP613216	OP723661
‘DahuacuipanJinwei’	‘Amabilis’ Group	75–156	Middle period (July and August)	Purple	Shaoyang, Hunan	OP613217	OP723662

## Data Availability

The plastome and nrDNA sequences under this study are deposited in the GenBank database under the following accession numbers: OP613198–OP613217 and OP723643–OP723662. The sequenced raw data are deposited in the SRA database with the accession number PRJNA909618.

## Supplementary Materials

The following supporting information can be downloaded at: https://www.mdpi.com/article/10.3390/ijms24043606/s1.
