# Supplementary figures and images for: Maternal Donor and Genetic Variation of Lagerstroemia indica Cultivars
Source: Int J Mol Sci. 2023 Feb 10;24(4):3606. doi: 10.3390/ijms24043606 (PMC9964644; doi:10.3390/ijms24043606)

**a**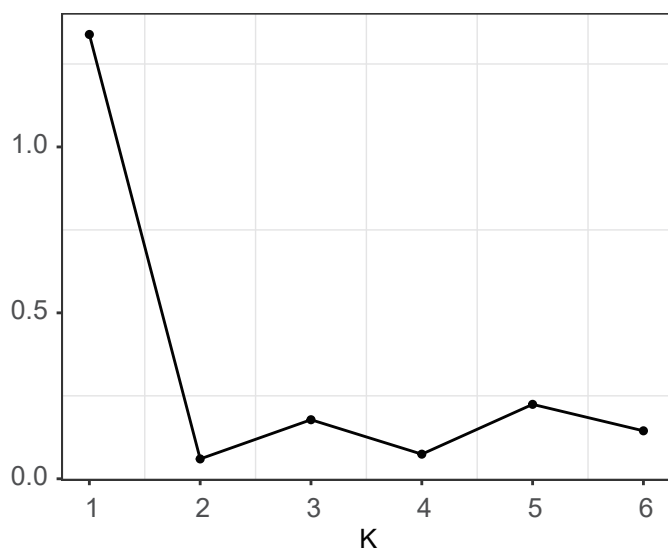**b**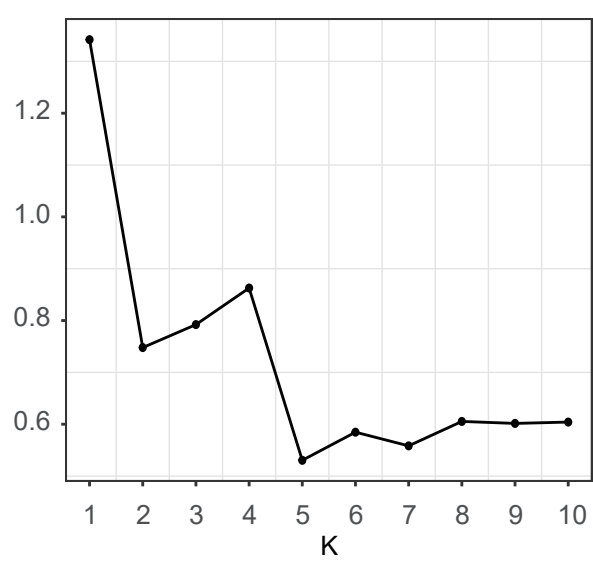

Supplement: Supplementary file 1 [file ijms-24-03606-s001.zip › Figure S1cv.pdf]
